# Scalability and internal consistency of the German version of the dementia-specific quality of life instrument QUALIDEM in nursing homes – a secondary data analysis

**DOI:** 10.1186/1477-7525-11-91

**Published:** 2013-06-05

**Authors:** Martin Nikolaus Dichter, Olga Dortmann, Margareta Halek, Gabriele Meyer, Daniela Holle, Johanna Nordheim, Sabine Bartholomeyczik

**Affiliations:** 1 German Center for Neurodegenerative Diseases (DZNE), Stockumer Straße 12, 58453 Witten, Germany; 2 School of Nursing Science, Witten/Herdecke University, Stockumer Straße 12, 58453 Witten, Germany; 3 Institute of Medical Sociology, Charité-Universitätsmedizin Berlin, Berlin, Germany

**Keywords:** Dementia, Scalability, Internal consistency, Quality of life, Nursing homes

## Abstract

**Background:**

Quality of life (Qol) is a widely selected outcome in intervention studies. The *QUALIDEM* is a dementia-specific Qol-instrument from The Netherlands. The aim of this study is to evaluate the scalability and internal consistency of the German version of the *QUALIDEM*.

**Methods:**

This secondary data analysis is based on a total sample of 634 residents with dementia from 43 nursing homes. The *QUALIDEM* consists of nine subscales that were applied to a subsample of 378 people with mild to severe dementia and six consecutive subscales that were applied to a subsample of 256 people with very severe dementia. Scalability, internal consistency and distribution scores were calculated for each predefined subscale using the Mokken scale analysis.

**Results:**

In people with mild to severe dementia, seven subscales, *care relationship, positive affect, negative affect, restless tense behavior, positive self-image, social relations* and *feeling at home,* were scalable (0.31 ≤ *H* ≤ 0.65) and internally consistent (Rho ≥ 0.62). The subscales *social isolation* (*H* = 0.28) and *having something to do* (*H* = 0.18) were not scalable and exhibited insufficient reliability scores (Rho ≤ 0.53). For people with very severe dementia, five subscales, *care relationship*, *positive affect*, *restless tense behavior*, *negative affect* and *social relations,* were scalable (0.33 ≤ H ≤ 0.65), but only the first three of these subscales showed acceptable internal consistency (Rho 0.59 – 0.86). The subscale *social isolation* was not scalable (*H* = 0.20) and exhibited poor internal consistency (Rho = 0.42).

**Conclusions:**

The results show an acceptable scalability and internal consistency for seven *QUALIDEM* subscales for people with mild to severe dementia and three subscales for people with very severe dementia. The subscales *having something to do* (mild to severe dementia), *negative affect* (very severe dementia), *social relations* (very severe dementia) and *social isolation* (both versions) produced unsatisfactory results and require revision.

## Background

The main goal of caring for people with dementia is the maintenance and promotion of their quality of life (Qol) [1]. Qol has become an important concept as an outcome in intervention studies, particularly psychosocial interventions, as well as an indicator of the quality of care of people with dementia [2-5]. The World Health Organization defines Qol as “*individuals’ perceptions of their position in life in the context of the culture and value systems in which they live and in relation to their goals, expectations, standards and concerns*” [6]. One early and highly recognized model describes dementia-specific Qol consisting of objective (e.g., behavioral competence and environment) and subjective (e.g., perceived Qol and psychological well-being) components (called ‘sectors’ by Lawton) [7,8]. Based on this theoretical approach, Jonker et al. developed a hierarchical model that defines psychological wellbeing as the starting point and central indicator for dementia-specific Qol [9]. Those authors argue for the consideration of non-dementia-related domains of Qol, such as personal factors (e.g., religion, income, age), next to environmental characteristics and dementia related domain.

In 2005, Ettema et al. defined the Qol of people with dementia based on a literature review that specified dementia-specific Qol as ‘*the multidimensional evaluation of the person-environment system of the individual, in terms of adaption to the perceived consequences of the dementia*’ [10]. Based on this definition, the seven adaptive tasks of the adaption coping model were interpreted as dementia-specific Qol domains: [11]*dealing with own disability, developing an adequate care relationship with the staff, preserving an emotional balance, preserving a positive self-image, preparing for an uncertain future, developing and maintaining social relationships* and *dealing with the nursing home environment*[12]. This model highlights the importance of psychosocial domains, which is supported by a recent review which showed 10 psychosocial (e.g., attachment, social contact, spirituality) as well as 3 physical and practical domains (e.g., physical health, financial situation) of Qol judged by people with dementia [13]. During the course of the theoretical developments, several dementia-specific Qol instruments have been developed, using self-ratings, proxy-ratings or direct observations as the data sources [14,15].

The majority of these instruments have been developed in English-speaking countries (particularly the USA and the UK). In Germany, Qol has recently been characterized as a nursing outcome by the medical service of the statutory long-term care insurance program. [16]. Only one German Qol instrument has been developed to date: the Heidelberg instrument for the assessment of quality of life in dementia (H.I.L.D.E.) [17]. This instrument is not typically applicable in research studies because it is moderately time-consuming (> 30 min per resident). A recent review did not identify any Qol instrument for people with dementia that has been validated in Germany [18]. To the best of our knowledge, there are a limited number of Qol instruments that have been translated into German, including the *Qol-AD *[19], *D-Qol *[20,21] and *QUALIDEM *[22]. These instruments have not been fully psychometrically tested. With the exception of the *QUALIDEM,* these instruments do not sufficiently focus on the Qol domains that are judged important for people with dementia [23].

The *QUALIDEM* has been evaluated in terms of psychometric properties with a focus on the psychosocial domains of dementia-specific Qol [12]. The instrument is simple to administer [24] and was developed for proxy-rating of Qol throughout the entire course of dementia in nursing home residents [25]. Consequently, the use of the *QUALIDEM* is recommended for Qol assessment in the late stage of disease [5] and for longitudinal ratings [26]. Its focus on psychosocial domains allows the instrument to assess several important Qol domains (affect, attachment, self-image, being useful, social contact, sense of aesthetics in the living environment, security and privacy, self-determination and freedom) that were described in an earlier review [13] and judged as important by people with dementia.

The *QUALIDEM* was developed and validated between 2005 and 2007 in the Netherlands. It consists of two consecutive versions for people with mild to severe and very severe dementia. The stages of dementia severity are classified according to the Reisberg scale, the Global Deterioration Scale (GDS) and Functional Assessment Staging (FAST), the last of which ranges from 1 (no cognitive impairment) to 7 points (very severe dementia) [27].

a) The Qol of people with mild to severe dementia (FAST = 2–6) is assessed by the 37-item version covering nine domains: *care relationship, positive affect, negative affect, restless tense behavior, positive self-image, social relation, social isolation, feeling at home* and *having something to do*.

b) The domains *positive self-image*, *feeling at home* and *having something to do* cannot be assessed in people with very severe dementia (FAST = 7). The second version of the *QUALIDEM* comprises 18 items covering six domains of Qol: *care relationship, positive affect, negative affect, restless tense behavior, social relation* and *social isolation*.

The response options for all items are “never”, “rarely”, “sometimes” and “frequently”. In 2008, the QUALIDEM was translated to German by a certified agency using forward-backward translation. The back-translated version was verified by the questionnaire’s first author, whose comments were taken into account for the adaption of the German version. In an exploratory investigation, the German QUALIDEM indicates construct validity measured by factor analysis and moderate to high internal consistency [22].

This paper outlines the evaluation of scalability (construct validity) and internal consistency of the German *QUALIDEM*, based on a large sample. The study followed a confirmatory methodological approach that has been used successfully by other studies in The Netherlands [12,26]. Additionally, the distribution of the subscales scores as differentiated by the subgroups of dementia severity, age and gender will be presented.

## Methods

A secondary data analysis of three German studies was performed. The data were collected from a pre/post-test evaluation of quality instruments in nursing homes (InDemA: Interdisciplinary Implementation of Quality Instruments for the Care of residents with Dementia in Nursing Homes) [28,29], a cluster-randomized controlled trial of the evaluation of the Serial Trial Intervention (STI-D: Serial Trial Intervention-Germany) [30] and a cross-sectional study on Dementia Care Mapping utilization (Leben-QD I: Strengthening Qol for people with dementia) [31].

The ethical committee of the German Society of Nursing Science approved the study protocol of the Qol-Dem project. Guidelines for the good practice of secondary data analysis AGENS [32] were applied for the quality assurance of data. Prior to data pooling, all three primary data sets were tested with respect to structure, completeness and plausibility. We also tested the comparability of the designs, measurements and samples of the three primary studies based on a systematic approach to pooled datasets of observational studies [33]. We judged differences between the three datasets as not likely to be relevant for our analysis.

### Setting and participants

Data collection took place between October and December 2008 for the InDemA study, between January and March 2009 for the STI-D study and between September and November 2010 for the Leben-QD I study. The total sample comprises 634 residents with dementia from 43 nursing homes located in the area of Frankfurt/Main (STI-D study, n = 19) and in North-Rhine Westphalia (InDemA study = 15, Leben-QD I study = 9).

The inclusion criteria for the residents were the following: Mini Mental Status Examination (MMSE) [34] score ≤ 24 (InDemA and STI-D) or a (FAST) [27] score ≥ 2 (Leben-QD I); living in the nursing home for at least 2 weeks (Leben-QD I and InDemA) or 4 weeks (STI-D). The exclusion criterion was a documented diagnosis of schizophrenia or other psychotic disorders (InDemA and STI-D).

### Procedures

Caregivers with different formal qualifications (registered nurses and nursing assistants) retrospectively filled in the questionnaires, with the answers referring to the last two weeks of observation. The caregivers were highly involved in the care of the people with dementia. To ensure standardization, the data collection was initiated by trained external research assistants (registered nurses and students in health care study programs).

### Measurements

In the InDemA and STI-D studies, the Mini Mental Status Examination (MMSE) was used for the assessment of the dementia severity. The MMSE ranges from 1 to 30 points (≤ 24 points: mild dementia, ≤ 10 points: severe dementia) [27]. The MMSE was given during an interview with the nursing home residents. Because this was associated with stress for many residents, the test was concluded early for ethical reasons if it was obvious that the resident would only reach an MMSE value < 10. Because there were no FAST scores available for the participants of the InDemA and STI-D studies (in contrast to Leben-QD I), the FAST scores of these participants were determined on the basis of their MMSE scores to form the two subsamples of interest (mild to severe dementia and very severe dementia). This approach followed a recommendation by Reisberg [27]. The different FAST stages were classified as follows: MMSE > 29 = FAST 1–2; 29 ≤ MMSE ≥ 24 = FAST 3; 23 ≤ MMSE ≥ 18 = FAST 4; 17 ≤ MMSE ≥ 14 = FAST 5, 13 ≤ MMSE ≥ 10 = FAST 6; and MMSE < 10 = FAST 7.

In all studies, the activities of daily living were assessed with the Physical Self Maintenance Scale (PSMS) [35]. This instrument consists of six items (toileting, feeding, dressing, grooming, physical ambulation and bathing). The response options range from 1 = no impairment to 5 = severe impairment, resulting in a total range of 6 to 30 points.

For the description of residents’ care dependency, levels defined by the German statutory Long-term Care Insurance were used (ranging from 1 = low to 3 = high). The sample characteristics of age and gender were assessed with single items.

### Statistical analysis

The Mokken scale analysis is a non-parametric iterative method for identifying unidimensional sets of polytomous items from a multidimensional item bank. It provides additional information about the relationship between items. The Mokken scale analysis is a method of item response theory (IRT), which is based on the following assumptions: unidimensionality, local independency and monotonicity. The method evolved from the Guttman analysis for investigating hierarchies of items within scales [36]. In contrast to the parametric Rasch analysis, the Mokken scale analysis requires no logistic function of an item as an assumption [37]. Because the ‘true’ course of an item trace-line is only visible after repeated investigation in several studies [38], we selected the Mokken scale analysis for the investigation of the ordinal *QUALIDEM* data. The Mokken scale analysis is established in the context of scale development, which has been widely used in nursing [39,40], psychology [41,42] and quality of life research [12,26,43,44]. The evaluation of a scale using Mokken scaling results in Loevinger’s coefficient *H* as an indicator for the scalability of each subscale. The scalability of a single item in relation to the other items in the scale or an item set is expressed by the value *H*_*i*_. According to Watson et al. [36], the following interpretation of *H* scores is applied: 0.30 – 0.39 = weak scale, 0.40 – 0.50 = medium scale, and > 0.50 strong scale. *H*_*i *_scores for each item should be non-negative for the Mokken model to hold. Additionally, the *H*_*i *_values should be > 0.30. Items that fail these *H*_*i *_levels have weak discrimination power and are not scalable [45].

Mokken’s confirmatory scalability analysis was used to investigate whether the subscales of the original Dutch version of the *QUALIDEM* are represented in the German data. Based on the existing subscales, Loevinger’s *H*_*i *_was calculated for each item of an item set, and *H* was calculated for each subscale. These calculations were performed for each of the three primary studies and for the total sample using the Mokken package for the software R [46,47]. The robustness of the scale is tested on different subpopulations [37].

The internal consistency of the *QUALIDEM* was assessed with the coefficient Rho. This coefficient is not as prone to bias as Cronbach’s alpha [48]. A Rho score > 0.60 indicates a reliable scale [49]. For comparison purposes only, we also calculated Cronbach’s alpha.

To investigate the distribution of the *QUALIDEM* scores, the 10th, 25th, 50th (median), 75th and 90th percentiles were calculated for each subscale for people with mild to severe dementia and for those with very severe dementia, grouped by gender and age. The subscale scores were transformed to values between 0 and 100. Cronbach’s alpha, percentile, means, standard deviations and the missing value analysis were performed using SPSS version 18.

## Results

### Study population

The sample consisted of 149 participants from the InDemA study, 338 participants from the STI-D study and 147 participants from the Leben-QD I study. For the investigation of both *QUALIDEM* versions, the sample was divided in two subsamples of 378 people with mild to severe dementia and 256 people with very severe dementia (Tables 1 and 2).

**Table 1:** Characteristics of the participants with mild to severe dementia (n = 378)

**Characteristics**	**InDemA**	**STI-D**	**Leben-QD I**	**Total**
	**n = 59 (15.6%)**	**n = 222 (58.7%)**	**n = 97 (25.7%)**	**n = 378 (100%)**
Age in years, mean±SD	82.3 ± 10.4	87.0 ± 7.6	85.2 ± 6.2	85.8 ±8.0
Female, n (%)	41 (69.5)	174 (78.4)	82 (84.5)	297 (78.6)
PSMS, mean±SD	18.1 ± 4.9	18.1 ± 4.9	17.3 ± 5.5	17.9 ±5.0
Care dependency levels^a^, n (%)				
None	3 (5.1)	4 (1.8)	5 (5.1)	12 (3.2)
1	29 (49.2)	67 (30.2)	34 (35.1)	130 (34.4)
2	26 (44.0)	113 (50.9)	48 (49.5)	187 (49.5)
3	1 (1.7)	38 (17.1)	10 (10.3)	49 (12.9)
FAST Score^b^, n (%)				
2	0 (0)	0 (0)	3 (3.1)	3 (0.8)
3	1 (1.7)	0 (0)	0 (0)	1 (0.3)
4	14 (23.7)	3 (1.4)	8 (8.2)	25 (6.6)
5	23 (39.0)	2 (0.9)	7 (7.2)	32 (8.5)
6	21 (35.6)	217 (97.7)	79 (81.5)	317 (83.9)
Missing Values *QUALIDEM*^c^	2/2183	10/8214	9/3589	19/13986

^a^As determined by expert raters of the medical service of the statutory long-term care insurance.

^b^The different FAST scores were classified as follows: *MMSE > 29 = FAST 1–2; 29 ≤ MMSE ≥ 24 = FAST 3; 23 ≤ MMSE ≥ 18 = FAST 4; 17 ≤  MMSE ≥ 14 = FAST 5; 13 ≤ MMSE ≥ 10 = FAST 6; and MMSE < 10 = FAST 7.*

^c^Each 37 *QUALIDEM* items per participant.

**Table 2:** Characteristics of the participants with very severe dementia (n = 256)

**Characteristics**	**InDemA**	**STI-D**	**Leben-QD I**	**Total**
	**n = 90 (35%)**	**n = 116 (45%)**	**n = 50 (20%)**	**n = 256 (100%)**
Age in years, mean±SD	84.2 ± 7.7	85.7 ± 8.4	82.0 ± 8.1	84.5 ± 8.2
Female, n (%)	71 (78.9)	89 (76.7)	39 (78.0)	199 (77.7)
PSMS, mean±SD	22.4 ± 3.8	24.3 ± 3.0	23.6 ± 2.9	23.5 ± 3.4
Care dependency levels^a^, n (%)				
None	1 (1)	0 (0)	0 (0)	1 (0.4)
1	17 (18.9)	8 (6.9)	4 (8.0)	29 (11.3)
2	40 (44.4)	47 (40.5)	19 (38,0)	106 (41.4)
3	32 (35.6)	61 (52.6)	27 (54,0)	120 (46.9)
Missing Values *QUALIDEM*^b^	1/1620	4/2088	8/900	13/4608

^a^As determined by expert raters of the medical service of the statutory long-term care insurance.

^b^Each 18 *QUALIDEM* items per participant.

### Missing value analysis

Of the 37 *QUALIDEM* items for people with mild to severe dementia, 19 responses (0.1%) from a maximum of 13986 possible responses were missing in the total sample (Table 1). Approximately 0.3% of responses were missing from the total sample of people with very severe dementia (Table 2). Based on the *two way imputation method* suggested by van der Ark and Sijtsma [50], the missing values were imputed for the next steps of the analysis.

### Scalability

The results are presented in Table 3. In brief, the results for the total sample of people with mild to severe dementia exhibited scalability of the adopted subscales with the exception of *social isolation* (*H* = 0.28) and *having something to do* (*H* = 0.18). Overall, the *H* values were stable in relation to the scalability level between the three primary studies. With the exception of the items *13, Indicates that he or she is bored*, *20, Openly rejects contact with others*, *26, Finds things to do without help from others*, *32, Calls out,* and *38, Enjoys helping with chores on the ward*, all the items in the adopted subscales were all scalable.

**Table 3:** **Scalability and internal consistency from nine *QUALIDEM *subscales for people with mild to severe dementia**

**FAST 2 - 6**		**InDemA**			**STI-D**			**Leben-QD I**			**Total**		
		**(n = 59)**			**(n = 222)**			**(n = 97)**			**(n = 378)**		
**Item Nr.**	**Subscale (items)**	**Scale-H**^**1 **^**(item H**_**i**_**)**	**Rho**	**Alpha**	**Scale-H**^**1 **^**(item H**_**i**_**)**	**Rho**	**Alpha**	**Scale-H**^**1 **^**(item H**_**i**_**)**	**Rho**	**Alpha**	**Scale-H**^**1 **^**(item H**_**i**_**)**	**Rho**	**Alpha**
**A.**	**Care relationship**	**0.51**	0.87	0.85	**0.41**	0.80	0.80	**0.41**	0.81	0.80	**0.42**	0.81	0.81
4.	Rejects help from nursing assistants	0.61			0.48			0.40			0.48		
7.	Is angry	0.57			0.50			0.44			0.49		
14.	Has conflicts with nursing assistants	0.61			0.49			0.54			0.52		
17.	Accuses others	0.39			0.32			0.29			0.32		
24.	Appreciates help he or she receives	0.53			0.37			0.35			0.39		
31.	Accepts help	0.55			0.41			0.43			0.43		
33.	Criticizes the daily routine	0.29			0.29			0.40			0.31		
**B.**	**Positive Affect**	**0.77**	0.94	0.93	**0.65**	0.91	0.91	**0.60**	0.88	088	**0.65**	0.91	0.90
1.	Is cheerful	0.79			0.70			0.66			0.67		
5.	Radiates satisfaction	0.79			0.71			0.60			0.69		
8.	Is capable of enjoying things in daily life	0.80			0.59			0.63			0.62		
10.	Is in a good mood	0.81			0.73			0.65			0.71		
21.	Has a smile around the mouth	0.75			0.67			0.62			0.66		
40.	Mood can be influenced in positive sense	0.69			0.52			0.43			0.52		
**C.**	**Negative Affect**	**0.48**	0.68	0.65	**0.58**	0.76	0.75	**0.43**	0.65	0.62	**0.53**	0.73	0.72
6.	Makes an anxious impression	0.47			0.51			0.40			0.49		
11.	Is sad	0.56			0.64			0.47			0.59		
23.	Cries	0.40			0.57			0.43			0.52		
**D.**	**Restless tense behavior**	**0.42**	0.68	0.66	**0.43**	0.70	0.66	**0.53**	0.76	0.74	**0.45**	0.69	0.68
2.	Makes restless movements	0.46			0.48			0.60			0.51		
19.	Is restless	0.45			0.50			0.60			0.51		
22.	Has tense body language	0.34			0.29			0.37			0.32		
**E.**	**Positive self-image**	**0.27**	0.55	0.49	**0.47**	0.70	0.71	**0.40**	0.64	0.63	**0.42**	0.67	0.67
27.	Indicates he or she would like more help	0.20			0.41			0.33			0.36		
35.	Indicates not being able to do anything	0.40			0.52			0.54			0.50		
37.	Indicates feeling worthless	0.23			0.46			0.34			0.41		
**F.**	**Social relations**	**0.57**	0.83	0.80	**0.41**	0.74	0.72	**0.38**	0.70	0.70	**0.43**	0.77	0.73
3.	Has contact with other residents	0.65			0.46			0.40			0.47		
12.	Responds positively when approached	0.47			0.48			0.31			0.44		
18.	Takes care of other residents	0.55			0.35			0.47			0.42		
25.	Cuts himself/herself off from environment	0.57			0.29			0.22			0.33		
29.	Is on friendly terms with one or more residents	0.57			0.42			0.42			0.45		
34.	Feels at ease in the company of others	0.57			0.48			0.46			0.48		
**G.**	**Social Isolation**	**0.12**	0.28	0.26	**0.30**	0.55	0.54	**0.29**	0.52	0.50	**0.28**	0.53	0.52
16.	Is rejected by other residents	0.21			0.39			0.41			0.35		
20.	Openly rejects contact with others	0.16			0.33			0.17			0.29		
32.	Calls out	−0.02			0.20			0.29			0.21		
**H.**	**Feeling at home**	**0.35**	0.68	0.64	**0.30**	0.59	0.60	**0.35**	0.64	0.63	**0.31**	0.62	0.61
13.	Indicates that he or she is bored	0.39			0.25			0.23			0.26		
28.	Indicates feeling locked up	0.26			0.33			0.44			0.34		
36.	Feels at home on the ward	0.34			0.32			0.28			0.30		
39.	Wants to get off the ward	0.40			0.30			0.43			0.34		
**I.**	**Having something to do**	**0.28**	0.37	0.36	**0.18**	0.18	0.23	**0.14**	0.24	0.22	**0.18**	0.23	0.24
26.	Finds things to do without help from others	0.28			0.18			0.14			0.18		
38.	Enjoys helping with chores on the ward	0.28			0.18			0.14			0.18		

^1^in bold.

Scale-H, Loevingers coefficient of homogeneity and scalability are classified as follow: 0.30 – 0.39 indicates a weak scale, 0.40 – 0.50 indicates moderate scalability and > 0.50 indicates strong scale.

The analysis for the people with very severe dementia demonstrated scalability for all predefined subscales excluding *social isolation* (*H* = 0.20). Between the primary studies, the difference in the *H* values varied from 0.11 for *restless tense behavior* and *social isolation* to 0.36 for *negative affect*. Items *16, Is rejected by other residents*, *20, Openly rejects contact with others*, *22, Has tense body language* and *32, Calls out,* were not scalable (Table 4).

**Table 4:** **Scalability and internal consistency from six *QUALIDEM *subscales for people with very severe dementia**

**FAST = 7**		**InDemA**			**STI-D**			**Leben-QD I**			**Total**		
		**(n = 90)**			**(n = 116)**			**(n = 50)**			**(n = 256)**		
**Item Nr.**	**Subscale (items)**	**Scale-H**^**1 **^**(item H**_**i**_**)**	**Rho**	**Alpha**	**Scale-H**^**1 **^**(item H**_**i**_**)**	**Rho**	**Alpha**	**Scale-H**^**1 **^**(item H**_**i**_**)**	**Rho**	**Alpha**	**Scale-H**^**1 **^**(item H**_**i**_**)**	**Rho**	**Alpha**
**A.**	**Care relationship**	**0.60**	**0.78**	**0.75**	**0.38**	**0.53**	**0.57**	**0.60**	**0.80**	**0.73**	**0.47**	**0.73**	**0.67**
7.	Is angry	0.60			0.50			0.61			0.54		
14.	Has conflicts with nursing assistants	0.66			0.44			0.63			0.54		
31.	Accepts help	0.53			0.19			0.53			0.35		
**B.**	**Positive affect**	**0.57**	**0.81**	**0.81**	**0.64**	**0.86**	**0.86**	**0.79**	**0.90**	**0.91**	**0.65**	**0.86**	**0.85**
5.	Radiates satisfaction	0.66			0.68			0.86			0.70		
8.	Is capable of enjoying things in daily life	0.52			0.67			0.75			0.64		
21.	Has a smile around the mouth	0.60			0.64			0.74			0.65		
40.	Mood can be influenced in positive sense	0.51			0.57			0.81			0.59		
**C.**	**Negative affect**	**0.38**	**0.50**	**0.49**	**0.46**	**0.62**	**0.57**	**0.10**	**0.25**	**0.15**	**0.36**	**0.50**	**0.47**
6.	Makes an anxious impression	0.38			0.46			0.10			0.36		
23.	Accepts help	0.38			0.46			0.10			0.36		
**D.**	**Restless tense behavior**	**0.41**	**0.66**	**0.66**	**0.32**	**0.57**	**0.56**	**0.43**	**0.73**	**0.66**	**0.37**	**0.59**	**0.62**
2.	Makes restless movements	0.53			0.37			0.56			0.47		
19.	Is restless	0.49			0.43			0.45			0.45		
22.	Has tense body language	0.21			0.14			0.27			0.18		
**F.**	**Social relations**	**0.33**	**0.58**	**0.53**	**0.41**	**0.59**	**0.58**	**0.10**	**0.18**	**0.19**	**0.33**	**0.53**	**0.52**
3.	Has contact with other residents	0.33			0.46			0.18			0.36		
12.	Responds positively when approached	0.28			0.41			0.14			0.34		
25.	Cuts himself/herself off from environment	0.37			0.36			0.00			0.30		
**G.**	**Social isolation**	**0.20**	**0.42**	**0.41**	**0.17**	**0.36**	**0.36**	**0.28**	**0.47**	**0.51**	**0.20**	**0.42**	**0.41**
16.	Is rejected by other residents	0.26			0.20			0.41			0.25		
20.	Openly rejects contact with others	0.18			0.24			0.22			0.21		
32.	Calls out	0.16			0.08			0.20			0.13		

^1^in bold.

*Scale-H*, Loevingers coefficient of homogeneity and scalability are classified as follow: 0.30 – 0.39 indicates a weak scale, 0.40 – 0.50 indicates moderate scalability and > 0.50 indicates strong scale.

### Internal consistency

The scalable subscales for mild to severe dementia exhibited a Rho coefficient between 0.62 and 0.91, which indicates internally consistent scales (Table 3). For people with very severe dementia, the scalable subscales of *care relationship*, *positive affect* and *restless tense behavior* were internally consistent (Rho ≥ 0.59) (Table 4).

### Percentile scores

The results in Table 5 demonstrate the percentage of people who have a Qol score above or below a certain score on a respective subscale. A higher score indicates a higher Qol.

**Table 5:** **Distribution of the *QUALIDEM *subscales for people with mild to severe and very severe dementia**

**Subscales, 0-100**^**a**^			**N**	**No. of Items**	**Mean (SD)**	**Percentiles**				
						**10%**	**25%**	**50%**	**75%**	**90%**
**A**	**Care relationship**									
	Mild to severe		378		75.1 (± 21.4)	43	62	81	95	100
	Age, years	≤ 80	77	7	74.7 (± 22.3)	38	62	81	93	100
		81 – 90	205		75.4 (± 20.4)	48	62	81	90	100
		> 90	96		74.7 (± 22.8)	41	57	81	95	100
	Gender	Female	297		75.9 (± 21.0)	47	62	81	95	100
		Male	81		71.8 (± 22.4)	35	57	76	90	100
	Very severe		256		75.2 (± 23.3)	44	56	78	100	100
	Age, years	≤ 80	69		68.9 (± 25.5)	33	56	67	89	100
		81 – 90	129	3	76.6 (± 22.8)	44	56	78	100	100
		> 90	58		79.7 (± 20.2)	54	67	78	100	100
	Gender	Female	199		76.5 (± 22.7)	44	67	78	100	100
		Male	57		70.6 (± 24.8)	33	56	67	94	100
**B.**	**Positive Affect**									
	Mild to severe		378		73.1 (± 23.5)	39	56	75	94	100
	Age, years	≤ 80	77		70.3 (± 24.5)	33	53	72	94	100
		81 – 90	205	6	74.7 (± 23.2)	39	61	78	97	100
		> 90	96		72.0 (± 23.2)	39	56	75	94	100
	Gender	Female	297		74.7 (± 22.6)	29	44	72	89	100
		Male	81		67.4 (± 25.7)	39	61	78	94	100
	Very severe		256		64.2 (± 27.5)	25	50	67	90	100
	Age, years	≤ 80	69		62.7 (± 28.9)	25	42	67	92	100
		81 – 90	129	4	63.7 (± 27.2)	17	50	67	83	100
		> 90	58		67.2 (± 26.7)	25	50	75	92	100
	Gender	Female	199		65.1 (± 27.0)	25	50	67	92	100
		Male	57		61.3 (± 29.3)	17	38	67	88	100
**C.**	**Negative Affect**									
	Mild to severe		378		71.4 (± 23.5)	44	56	78	89	100
	Age, years	≤ 80	77		72.2 (± 24.4)	33	56	78	89	100
		81 – 90	205	3	70.7 (± 23.7)	44	56	78	89	100
		> 90	96		72.3 (± 22.7)	44	56	78	89	100
	Gender	Female	297		69.1 (± 24.3)	33	56	67	89	100
		Male	81		80.0 (± 18.0)	56	67	89	89	100
	Very severe^b^		256		69.4 (± 27.7)	33	50	67	100	100
	Age, years	≤ 80	69		67.6 (± 29.4)	17	50	83	100	100
		81 – 90	129	2	68.0 (± 27.5)	17	50	67	100	100
		> 90	58		74.7 (± 25.8)	33	50	83	100	100
	Gender	Female	199		66.8 (± 28.0)	17	50	67	100	100
		Male	57		78.4 (± 24.8)	33	67	83	100	100
**D.**	**Restless tense behavior**									
	Mild to severe		378		66.6 (± 29.4)	22	44	67	89	100
	Age, years	≤ 80	77		66.5 (± 33.3)	11	33	78	100	100
		81 – 90	205	3	66.6 (± 29.0)	22	44	67	89	100
		> 90	96		66.9 (± 27.0)	33	44	67	89	100
	Gender	Female	297		66.6 (± 29.6)	22	44	67	89	100
		Male	81		66.8 (± 28.8)	22	44	78	89	100
	Very severe		256		55.9 (± 29.4)	11	33	56	78	100
	Age, years	≤ 80	69		49.4 (± 30.7)	0	22	56	67	100
		81 – 90	129	3	56.8 (± 29.3)	11	33	56	78	100
		> 90	58		61.7 (± 27.2)	22	33	67	81	100
	Gender	Female	199		57.4 (± 29.3)	11	33	56	78	100
		Male	57		50.9 (± 29.5)	11	22	56	78	89
**E.**	**Positive self-image**									
	Mild to severe		378		77.6 (± 25.7)	44	67	89	100	100
	Age, years	≤ 80	77		78.5 (± 25.6)	33	67	89	100	100
		81 – 90	205	3	76.9 (± 26.1)	40	67	89	100	100
		> 90	96		78.6 (± 25.1)	44	67	89	100	100
	Gender	Female	297		77.0 (± 25.9)	44	67	89	100	100
		Male	81		80.0 (± 24.8)	33	67	89	100	100
**F.**	**Social relations**									
	Mild to severe		378		64.7 (± 21.4)	33	50	67	83	89
	Age, years	≤ 80	77		63.6 (± 24.5)	32	44	67	83	94
		81 – 90	205	6	66.2 (± 20.5)	39	50	67	83	94
		> 90	96		62.3 (± 20.7)	33	44	67	78	89
	Gender	Female	297		66.6 (± 20.5)	39	50	67	83	94
		Male	81		57.4 (± 23.3)	23	44	61	75	89
	Very severe^b^		256		66.3 (± 25.7)	22	47	67	89	100
	Age, years	≤ 80	69		64.9 (± 25.6)	22	44	67	89	100
		81 – 90	129	3	64.9 (± 26.3)	22	56	67	89	100
		> 90	58		70.9 (± 24.3)	33	56	78	89	100
	Gender	Female	199		67.2 (± 24.8)	33	56	67	89	100
		Male	57		63.2 (± 28.6)	22	44	67	89	100
**G.**	**Social isolation**									
	Mild to severe^b^		378		73.7 (± 25.0)	33	56	78	100	100
	Age, years	≤ 80	77		79.2 (± 22.8)	44	61	89	100	100
		81 – 90	205	3	72.2 (± 25.5)	33	56	78	100	100
		> 90	96		72.2 (± 24.9)	33	56	78	89	100
	Gender	Female	297		73.4 (± 25.5)	33	56	78	100	100
		Male	81		74.5 (± 22.7)	44	56	78	100	100
	Very severe^b^		256		75.4 (± 23.3)	41	67	78	100	100
	Age, years	≤ 80	69		71.5 (± 24.1)	33	56	67	100	100
		81 – 90	129	3	77.9 (± 22.5)	44	67	78	100	100
		> 90	58		74.5 (± 23.7)	33	67	78	100	100
	Gender	Female	199		74.9 (± 23.4)	44	67	78	100	100
		Male	57		77.2 (± 23.1)	33	61	89	100	100
**H.**	**Feeling at home**									
	Mild to severe		378		78.0 (± 21.6)	42	67	83	100	100
	Age, years	≤ 80	77		78.0 (± 22.7)	42	67	83	100	100
		81 – 90	205	4	76.5 (± 22.2)	42	67	83	92	100
		> 90	96		81.2 (± 19.2)	50	67	92	100	100
	Gender	Female	297		79.0 (± 21.1)	50	67	83	100	100
		Male	81		74.5 (± 22.7)	42	58	75	92	100
**I**	**Having something to do**									
	Mild to severe^b^		378		40.4 (± 29.5)	0	17	50	50	83
	Age, years	≤ 80	77		44.2 (± 31.3)	0	17	50	67	100
		81 – 90	205	2	40.8 (± 29.3)	33	56	72	89	100
		> 90	96		36.6 (± 28.2)	33	56	78	100	100
	Gender	Female	297		42.5 (± 29.9)	0	17	50	67	83
		Male	81		32.9 (± 26.6)	0	0	33	50	67

^a^Subscale sum scores were transformed to values between 0 and 100 using the formula: QUALIDEM(%) = QUALIDEM(Sumscore)*100/3*N(number of items for each subscale). Higher scores indicate a higher Qol.

^b^Not scalable and unreliable subscale.

For all subscales, the median for participants was ≥ 50 regardless of the dementia severity, which reflects relatively high scores in general. The median in the subscales *care relationship* (81–78), *positive affect* (75–67), *negative affect* (78–67) and *restless tense behavior* (67–56) exhibited higher scores for people with mild to severe dementia than for people with very severe dementia. The Qol domains *social relations* and *social isolation* exhibited no difference in medians. The analysis of the 3 age groups demonstrated a stable distribution of the Qol scores for all subscales. With respect to gender, the median scores of men were higher in the subscales *positive affect*, *negative affect*, *restless tense behavior* and *social isolation* than the median scores of the women. In contrast, the subscales, *care relationship*, *social relations*, *feeling at home* and *having something to do* exhibited higher median scores for women.

## Discussion

The results for the 37-item version for mild to severe dementia revealed that 7 of 9 subscales were scalable and internally consistent. The *H* values were stable with respect to the scalability level, independent of the nursing institution or primary study. The subscales *social isolation* and *having something to do* were not scalable. These results are largely comparable to a previous Dutch study in which the subscale *social isolation* was not scalable and the subscale *having something to do* was weakly scalable [26]. Consistent with these results, the subscale, *social isolation,* could not be identified in the German version of the *QUALIDEM* through an explorative factor analysis [22]. The results for the subscale *social isolation* might be explained by item *32, Calls out*. Calling out is not necessarily an expression of social isolation, as it could be an expression of pain or stress without social isolation. The negative result for the subscale *having something to do* can be explained by the different meanings of the representing items. Item *26, Finds things to do without help from others,* assesses an ability (physical and/or cognitive), whereas Item *38, Enjoys helping with chores on the ward,* indicates a social behavior and mood/emotions. Item *13*, *indicating that he or she is bored,* was not scalable, whereas the corresponding subscale, *feeling at home,* was scalable. Previous studies revealed different results [12,22,26]. The internal consistency of all the scalable scales varied from 0.91 (very good) to 0.62 (acceptable) for the 37-item version.

The analysis for the 18-item version of the *QUALIDEM* revealed that only the three subscales *care relationship*, *positive affect* and *restless tense behavior* were scalable and internally consistent. The Rho scores for the mentioned subscales varied between 0.86 (good) and 0.59 (just acceptable). This result is consistent with previous Dutch studies [12,26] and the results for alternative Qol instruments [15]. The remaining subscales were not reliable (*negative affect* and *social relations*) and not scalable (*social isolation*). The *H* values varied between the different primary studies, depending on the subscale, and no pattern was discernible. The insufficient internal consistency for these three subscales [26] and the unsatisfactory scalability of the subscale, *social isolation,* is consistent with previous results [22,26]. The subscale, *negative affect,* consists of 2 items, and the subscale, *social relations*, consists of three items. This difference could be a reason for the weak internal consistency. With respect to the *H*_*i*_ values for each item, item 22, *has tense body language,* was not scalable. This finding is consistent with the results of Bouman et al. [26], who found a *H*_*i *_value of 0.29 for this item.

The investigation of distributional scores identified relatively high Qol scores in general. For all subscales, 50% of the participants reached a score of 50 or higher, regardless of dementia severity. This result raises the question of the QUALIDEM’s sensitivity for change, which has not been assessed. Information on responsiveness is scarce in general [51], which highlights the need for research on this topic. To use Qol as an outcome in intervention studies, evidence of the QUALIDEM’s sensitivity for change is required [52].

At the 50th percentile, people with mild to severe dementia exhibited higher Qol scores in the subscales *care relationship*, *positive affect*, *negative affect* and *restless tense behavior* than did people with very severe dementia. In the latest review investigating the influence of cognition on health-related quality of life (HRQL) in dementia, no convincing evidence was found that lower cognitive abilities are associated with a lower HRQL [53]. In a recent study, cognition was identified as a predictor of Qol [54]. Depending on the subscale, either men or women exhibited higher values at the 50th percentile. In the mentioned review, gender did not appear to have any effect on HRQL [53]. The differences in the Qol-distribution between different age and cognition groups and gender require further investigation for verification. In accordance with previous findings [54,55], our results of the descriptive statistical analyses did not exhibit substantial differences between age groups regarding Qol values.

The skewed distribution towards high Qol values raises the question of the validity of the data. The Qol assessment using the *QUALIDEM* is based on a proxy-assessment, which is preferred in advanced dementia and for longitudinal Qol evaluation [10]. Proxy-rated Qol instruments have several methodological difficulties. Proxy-rated Qol values from people with dementia are influenced by the burden [56] and attitudes of proxy-raters [57]. In several studies, the scores are systematically lower than self-rated Qol values [19,58]. Proxy-raters are professional caregivers who are responsible for the wellbeing of the residents and might underlie social desirability. According to Gerritsen et al. [59], Dutch caregivers as proxy-raters focus strongly on the majority of the domains considered important by people with dementia. The professional caregivers concentrate on the domains *affect*, *self-esteem*, *attachment*, *security and privacy*, *social contact* and *physical and mental health*. Little attention was paid to the domains *financial situation* and *being useful/giving meaning to life*. The consistency of QUALIDEM subscales and the Qol domains nurses focus on underpins the validity of the instrument. To what extent these results are transferable to Germany remains unclear because of the differing training programs for caregivers in Germany. The evidence of proxy-ratings appears to contradict the possible assumption that the reported high Qol values are affected by the perspective of the Qol assessment.

### Limitations

Our analysis is based on a large sample of people with dementia at all stages of the disease. Some sub-analyses are based on a small sample size of n = 50. The large correlation with previous findings with respect to the scalability and internal consistency of the adopted subscales supports the validity of the study results [12,26]. These results must be interpreted based on the existing reliability data from the Netherlands. A first Dutch investigation of interrater and intrarater reliability showed a moderate to strong agreement depending on the *QUALIDEM* subscales [12]. There are preliminary results for the subscales of the German *QUALIDEM* that are comparable to the mentioned investigation [60]. Based on the available data, the FAST scores of the participants of the two primary studies (InDemA and STI-D) were determined based on their MMSE scores. Contrary to a recommendation by Reisberg [27], the patients with an MMSE score < 10 instead of an MMSE value < 6 were assigned to the very severe dementia group. A few more participants may have been assigned to the group of people with very severe dementia. This difference should have no effect on the results of the scalability and internal consistency because the 18 items for very severe dementia were also assessable for this group. This different classification might be a potential bias for the distribution scores of the subscales. Other influences on these scores were not investigated (e.g., effect of the type of dementia).

## Conclusions

Our study demonstrates the acceptable scalability and internal consistency for seven subscales for people with mild to severe dementia and three subscales for people with very severe dementia exhibit robustness in different subpopulations. These results are largely consistent with previous findings. Concerning the subscales *having something to do* (mild to severe dementia), *negative affect* and *social relations* (both very severe dementia), the extent to which rewording of the items produces better scalability and internal consistency should be explored. The clarity and selectivity of the items of these subscales in particular must be taken into account, as the interpretation of the current items can differ. For example, item *12, responds positively when approached,* is not a precise item of the subscale *social relations*, as the wording of this item is similar to items from the subscale *positive affect*. This finding is emphasized by a high factor loading on *positive affect* in a previous exploratory study [22]. The subscale *social isolation* should be omitted in the future because it was neither scalable nor internally consistent and represents a duplication of the content in the subscale *social relations*.

A revision is recommended for item *13, indicating that he or she is bored,* which is not scalable in its present format. The presented percentile scores provide orientation values for comparisons with future studies. The relatively high Qol values for all subscales underline the need for further investigation of the *QUALIDEM*. Such investigations must be theory-based validity tests that will be part of the Qol-Dem study. Methodological questions such as the possible influence of proxy characteristics (e.g., burden and attitudes on dementia) and the decision-making process of proxies through the Qol assessment of people with dementia in general should be taken into account in future research. In the next steps of the Qol-Dem project, the inter-rater and intra-rater reliability and validity (criterion and construct) of the German version of the *QUALIDEM* will be comprehensively investigated. These studies will result in a broader understanding of the quality of the *QUALIDEM*, which could be used for the development of a more advanced version of the instrument.

## Competing interests

The authors’ declare that they have no competing interests.

## Authors’ contributions

Study design: MND, SB, MH, GM, Data collection and handling: MND, JN, DH, OD, Data analysis: MND, OD. Manuscript preparation: MND, OD, MH, GM, DH, JN, SB. All authors read and approved the final manuscript.
